# Pulmonary artery diameter and NT-proBNP in patients with Covid-19: Predicting prognosis and mortality

**DOI:** 10.4314/ahs.v23i2.64

**Published:** 2023-06

**Authors:** Mercan Tastemur, Esin Olcucuoğlu, Gunes Arik, Ihsan Ates, Kamile Silay

**Affiliations:** 1 Ministry of Health, Ankara City Hospital, Department of Geriatrics Medicine; 2 Ministry of Health, Ankara City Hospital, Department of Radiology; 3 Ministry of Health, Ankara City Hospital, Department of Internal Medicine

**Keywords:** COVID-19, Pulmonary artery diameter, MPAD/AAD ratio, NT-proBNP

## Abstract

**Background:**

The diverse and complex presentations of COVID-19 continue to impact the world. Factors related to prognosis and mortality are still not fully illuminated.

**Objectives:**

We aimed to asses the relationship of N-terminal pro B-type natriuretic peptide (NT-proBNP) and main pulmonary artery diameter (MPAD) with COVID-19 prognosis and mortality.

**Methods:**

152 COVID-19 patients over the age of 18, were included in the study. Thoracic CT, NT-proBNP values, laboratory and demographic data of these patients were obtained by retrospectively examining the patient files and scanning the results through the patient registry.

**Results:**

According to multivariate logistic regression (LR) analysis, high NT-proBNP level (OR=3.542; 95% CI=1.745-9.463; p=0.021) and MPAD/ascending aortic diameter (AAD) ratio>0.75 (OR=2.692; 95% CI=1.264-9.312; p=0.036) were determined as independent risk factors predicting mortality in COVID-19 patients. A significant positive correlation was observed between NT-proBNP level and MPA diameter (r=0.296, p<0.001). The cut-off value was measured as 27.5 mm for MPA diameter and 742 pg/ml for NT-proBNP.

**Conclusions:**

Accurate and effective interpretation of available radiological and laboratory data is essential to reveal the factors predicting prognosis and mortality in COVID-19. In this study,we evaluated that the thorax CTs and determined that the MPAD/AAD and NT-proBNP level were independent risk factors in predicting mortality.

## Introduction

The infectious disease caused by SARS-CoV2, which emerged in Wuhan, China in December 2019 and named as COVID-19 by the World Health Organization (WHO) in February 2020, has turned into a pandemic that affects the whole world. Since the onset of the disease, more than 300,000,000 cases have been confirmed worldwide, and the cumulative deaths exceed 5,000,000 1,2.

Although RT-PCR testing plays a crucial role in accurately detecting SARS-CoV-2 on a case-by-case basis, it also has issues that limit its usefulness. Obstacles to the widespread use of RT-PCR test are generally lack of test kits, laboratory requirement, long processing time until results, false negatives. However, given the high specificity and sensitivity of the RT-PCR test, it is concluded that it alone should be used as the primary diagnostic tool. This analysis is also supported by the recommendations made by the American College of Radiology during the COVID-19 outbreak 3. Early identification and isolation of COVID-19 patients are crucial in controlling this outbreak, especially in those with false-negative RTPCR or asymptomatic patients. Thoracic CT is not the primary examination in diagnosis. However, there are studies suggesting that CT can be used primarily as a complementary tool in patients with symptoms for more than 2 days, in symptomatic patients with negative RT-PCR test results, and in suspected patients. In COVID-19, thoracic CT findings may vary both as the disease progresses and from patient to patient. Therefore, it is thought to be useful in the evaluation of prognosis 4, 5.

In the appropriate setting, CT scanning can assist in the diagnosis of COVID-19, provide a basis for patients at risk for disease progression, and help ensure that alternative diagnosis and comorbidities are not missed in high-risk patients with COVID-19. It is also a tool for rapid triage in situations with limited resources and high disease burden. As disease and pandemic evolve, there may be more updates in the role of CT in disease management and control 6.

COVID-19 is characterized by an exaggerated inflammatory response that can lead to serious conditions such as acute respiratory distress syndrome (ARDS), sepsis, coagulation and death as a consequence. Coagulopathies have been reported in approximately 50% of patients with severe COVID-19 symptoms. Complications are related to thrombosis defined in many organs especially in pulmonary microvasculature 7. Autopsy studies indicate the presence of pulmonary endothelial damage and microthrombosis. Although thromboembolism is not observed in the main pulmonary arteries, small thrombi can be observed in the periphery of the lung parenchyma. Autopsy results also revealed that capillary endothelial and microvascular dysfunction may cause cardiac injury and ultimately cell necrosis 8.

Parenchymal imaging findings of COVID-19 pneumonia are well defined. Vascular enlargement, which occurs with inflammatory and vascular changes, is an important entity that causes high thromboembolic risk after pulmonary hemodynamic deterioration. Noncontract CT is a useful and non-invasive option for demonstrating vascular effects and measuring the MPA diameter and MPAD/AAD ratio. Such measurements, which may represent a potential prognostic index, are easily available in COVID-19 patients currently undergoing non-contrast CT scans 9, 10.

The pulmonary artery originates from the right ventricle above the pulmonary valve. In adults, the main pulmonary artery (MPA) is approximately 5 cm long and is completely enclosed within the pericardium. In the Framingham Heart Study, MPAD was determined as ≤29 mm in men and ≤27 mm in women, and MPAD/AAD ratio ≤0.9 in normal values, according to CT measurements 11 Pulmonary artery-to-aorta MPAD/AAD ratio is a ratio used for screening and evaluation of pulmonary hypertension. It has been suggested that this ratio being greater than 1 or 1.1 is suggestive of pulmonary hypertension 12. Pulmonary artery diameters are affected in various pulmonary pathologies. Pneumonia is one of the most common diseases among these pathologies. Studies in young and middle-aged patients have shown that pneumonia can increase pulmonary artery pressures depending on the degree of respiratory compromise and hypoxemia 13. In hypoxemic patients with acute pneumonia, adaptive local regulatory mechanisms may influence pulmonary vascular blood flow resistance to reduce consolidation of the lung parenchyma and pulmonary ventilation perfusion mismatches (ie shunts) 14. Pneumonia also affects the cardiovascular system by many mechanisms. Increased vascular resistance can cause depression of LV function, heart muscle inflammation, increased concentrations of troponin, BNP, and ANP, increased pulmonary artery pressures, and impaired cardiovascular autonomic reflexes 15.

Diffuse lung consolidation and ARDS may alter pulmonary vascular properties, causing pulmonary hypertension. Today, these processes are encountered in COVID-19 patients. Therefore, early detection of pulmonary hypertension is crucial to guide appropriate treatment. While non-contrast CT is sufficient for pulmonary artery diameter enlargement, contrast enhanced CT is more sensitive for thrombus diagnosis. However, the fact that this procedure requires iodinated contrast material may cause it to be a contraindication for COVID-19 patients 16.

Brain natriuretic peptide (BNP) was initially detected in brain tissue, but is known to be a natriuretic hormone primarily released from the heart, particularly the ventricles. Cleavage of the prohormone pro-BNP produces 32 biologically active amino acid BNP as well as the biologically inert 76 amino acid N-terminal pro-BNP (NT-proBNP). The main stimulus for NT-proBNP synthesis fand secretion from cardiac myocytes is myocyte stretch. In heart failure, increased wall tension, neurohormonal activation, and hypoxia stimulate BNP secretion 17. Although tension is considered the main stimulus for cardiac NT-proBNP secretion, some hormones (such as catecholamines, angiotensin II, and endothelin) may also be involved. Furthermore, recent studies suggest that hypoxia can be stimulatory independent of muscle tension 18. Apart from cardiac causes, sepsis, pulmonary hypertension, and surgical procedures may also cause an increase in NT-proB-NP. Increasing vascular resistance with the involvement of the lungs will especially affect the right heart and cause the release of NT-proBNP. Nagaya et al. showed that plasma BNP levels increase in pulmonary hypertension in proportion to the degree of RV dysfunction 19-21.

Hemodynamic stress caused by various reasons causes NT-proBNP levels to increase in laboratory results. Many studies suggest that NT-proBNP is frequently elevated in COVID19 patients and has a prognostic significance 22-26. This increase may mostly be due to possible right ventricular dilatation, or may be due to the virus itself 8.

A limited number of studies revealed the relationship between pulmonary artery diameter and the severity of COVID-19 disease. There are also studies showing that the diameter of the PA increases independently of the lung involvement of the disease, mostly due to microvascular pathologies 9, 10, 16, 27, 28.

Non-contrast CT examination can be performed in almost every patient to reveal the severity of the disease. In this study, we measured the pulmonary artery diameter from thoracic CT scans in patients followed up for COVID-19 in the internal medicine clinics of our hospital and evaluated them together with NT-proBNP levels. We evaluated the relationship between these findings and prognosis/ mortality of COVID-19.

## Methods

This study is a retrospective cohort study in which patients followed for COVID-19 in the internal medicine clinics of our tertiary hospital were included.

### Study Population

In our study, thoracic CT scans and NT-proBNP levels of 152 patients aged 18 years and older hospitalized in our internal medicine COVID-19 clinics between November and August 2020 were included. Patients with a known history of pulmonary hypertension and embolism were excluded from the study.

### Data Collection

Demographic data of the patients (age, gender, comorbid diseases), laboratory; hemogram, biochemistry, d-dimer, carp, procalcitonin, ferritin, interleukin-6, troponin-I, NTproBNP levels and data such duration of hospitalization, need for oxygen support, need of intensive care were recorded. Our study was approved by the Clinical Research Ethics Committee (Date: 28.102020, Decision: E1-20 1252). The research was carried out in accordance with the Declaration of Helsinki.

### CT Acquisition

All thoracic CT scans were performed with a 128-detector system (GE Revolution, General Electric, Milwaukee, WI) without contrast using the following parameters: 100 kV, 110 mAs, body filter, 1.25 mm slice thickness, 512x512 reconstruction matrix, spiral pitch factor 1.375: one.

### Image analysis

Our radiologist with 11 years of experience reviewed the CT scans to evaluate the pulmonary parenchyma, pulmonary vascular metrics and Ao maximum diameter.

Lung parenchyma was assessed for the presence of the following features: GGOs and/or consolidation, crazy-paving pattern, pleural effusion. Pneumonia grade was visually evaluated according to the categories suggested by Bernheim et al 29. Each of five lung lobes was assessed for degree of involvement, which was classified as none (0%), minimal (1%–25%), mild (26%–50%), moderate (51%–75%), or severe (76%–100%). No involvement group corresponded to a lobe score of 0, minimal to a lobe score of 1, mild to a lobe score of 2, moderate to a lobe score of 3, and severe to a lobe score of 4. An overall lung total severity score was reached by summing the five lobe scores 29. Finally, the PA maximum diameter at the level of its bifurcation and the Ascending aortic maximum diameter were assessed in a single section and MPAD/AAD ratio then calculated 30. (Picture 1)

**Figure:**
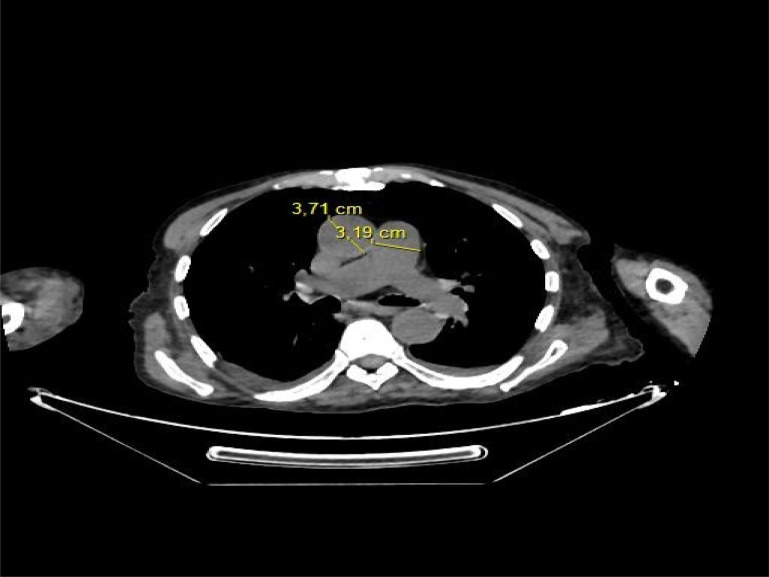
Picture 1

### Statistical Analysis

Data coding and statistical analysis were performed on the computer using the SPSS 22 software (IBM SPSS Statistics, IBM Corporation, Chicago, IL) package program. The conformity of the variables to the normal distribution was examined using the Shapiro-Wilk tests. Normally distributed variables were expressed as mean ± standard deviation, non-normally distributed variables were expressed as median (minimum-maximum) values. In the comparison of non-categorical parameters between the groups, Student's T test was used for those with normal distribution and Mann-Whitney U test for those who were not normally distributed. Chi-square and Fisher's exact tests were used for categorical variables. The predicting features of MPA diameter, MPAD/AAD ratio, and NT-proBNP level for mortality of COVID-19, were analysed with the ROC curve at 95% confidence interval. Risk factors for mortality in COVID-19 patients were determined by univariate logistic regression analysis.

Whether the possible factors identified in this analysis were independent risk factors, were evaluated using the Backward LR method with multivariate analysis. The correlation between the two variables was evaluated using the Pearson and Spearman correlation coefficients. Those with a p value below 0.05 were considered statistically significant.

## Results

The mean age of the 152 COVID-19 patients included in the study was 67.2±14 years. While 122 (80.3%) patients were in the convalescent group, 30 (19.7%) patients were in the deceased group. According to radiological findings; MPA diameter, ratio of MPAD/AAD and presence of pleural fluid were higher in the deceased group. Demographic data, clinical status, co-morbidities, laboratory results, treatment and radiological findings of the patients are shown in Table 1.

**Table 1:** Demographic, clinical, laboratory and radiological characteristics of patients who were recovered or death

	Total (n=152)	Recovered (n=122, 80.3%)	Death (n=30, % 19.7)	p
**Demographic data**				
Age (years) (Mean ± SD)	67.2±14	65.7±14	73.3±12.6	**0.003^m^**
Female gender, n (%)	73 (48)	56 (45.9)	17 (56.7)	0.29^x^
**Clinical data**				
Hospitalization time (days) (median)(min-max)	12 (2-41)	12 (2-41)	14.5 (2-40)	0.104^m^
Presence of oxygen demand, n (%)	98 (64.5)	72 (59)	26 (86.7)	**0.005^x^**
Intensive care requirement, n (%)	39 (25.7)	22 (18)	17 (56.7)	**<0.001^x^**
**Additional diseases**				
Hypertension, n (%)	93 (61.2)	69 (56.6)	24 (80)	**0.018^x^**
Diabetes Mellitus, n (%)	48 (31.6)	36 (29.5)	12 (40)	0.28^f^
COPD, n (%)	32 (21.1)	25 (20.5)	7 (23.3)	0.732^x^
CAD, n (%)	61 (40.1)	42 (34.4)	19 (63.3)	**0.004^x^**
Dementia, n (%)	9 (5.9)	3 (2.5)	6 (20)	**0.002^f^**
**Laboratory data**				
Albumin (g/dL) (Mean ± SD)	3.7±0.5	3.8±0.4	3.4±0.6	**0.019^t^**
LDH (U/L) (Median)(min-max)	343.5 (146-948)	337 (146-919)	376.5 (216-948)	0.055^m^
CRP ( gr/L) (median)(min-max)	60.5 (0.8-401)	52 (0.8-401)	106 (5.5-333)	**0.011^m^**
Fibrinogen (g/L) (Median)(min-max)	4.7 (1.4-445)	4.7 (2-445)	4.7 (1.4-8)	0.498^m^
D-dimer (mg/L) (median)(min-max)	0.9 (0.2-21)	0.8 (0.2-16.4)	1.6 (0.5-21)	**<0.001^m^**
Procalcitonin (µg/L) (median)(min-max)	0.1 (0.01-8.6)	0.1 (0.01-8.6)	0.1 (0.02-5)	**0.004^m^**
Maximum ferritin level (µg/L) (Median)(min-max)	595 (9-65356)	514 (9-11970)	1316 (125-65356)	**<0.001^m^**
Interleukin-6 (pg/mL) (median)(min-max)	30.3 (2-1000)	28.1 (2-1000)	38 (13-596)	**0.023^m^**
Maximum interleukin-6 level (pg/mL) (median)(min-max)	31.1 (2-9203)	27.1 (2-9203)	78 (14.2-2311)	**<0.001^m^**
NT-proBNP (pg/mL) (median)(min-max)	542 (35-23552)	451 (35-35000)	1748 (35-23552)	**0.001^m^**
Troponin (ng/mL) (median)(min-max)	11.5 (2-2073)	10.5 (2-1290)	15 (3-2073)	0.069^m^
**Radiological findings**				
MPAD (mm) (median)(min-max)	28.2 (20-58.4)	28 (20-52.8)	29.8 (24.2-58.4)	**0.036^m^**
AAD (mm) (Mean ± SD)	37±4.1	37±4.3	37.1±3.3	0.221^t^
MPAD/AAD ratio (median)(min-max)	0.8 (0.5-1.6)	0.8 (0.5-1.3)	0.8 (0.6-1.6)	**0.037^m^**
**CT spread finding**				
None	29 (19.1)	20 (16.4)	9 (30)	0.14^f^
Minimal	8 (5.3)	6 (4.9)	2 (6.7)	
Moderate	10 (6.6)	10 (8.2)	0 (0)	
Severe	105 (69)	86 (70.5)	19 (63.3)	
**Number of lobes involved**				
**None, n (%)**	29 (19.1)	20 (16.4)	9 (30.1)	0.308^f^
**1, n (%)**	3 (2)	2 (1.6)	1 (3.3)	
**2, n (%)**	5 (3.3)	4 (3.3)	1 (3.3)	
**3, n (%)**	9 (5.9)	9 (7.4)	0 (0)	
**4, n (%)**	6 (3.9)	5 (4.1)	1 (3.3)	
**5, n (%)**	100 (65.8)	82 (67.2)	18 (60)	

**COVID-19:** Coronavirus Disease 19 **COPD:** Chronic Obstructive Pulmonary Disease, **CAD:** coronary Artery disease **LDH:** Lactate dehydrogenase, **CRP:** C reactive protein, **IL-6:** Interleukin 6, **NT-proBNP:** N-terminal pro B-type natriuretic peptide **MPAD:** Main pulmonary artery diameter, **AAD:** Ascending aortic diameter **CT:** Computed Tomography, **m:** Mann Whitney U Test, **t:** Student't T Test x: Chi-square Test, **f:** Fisher's Exact Test

**Figure 1:**
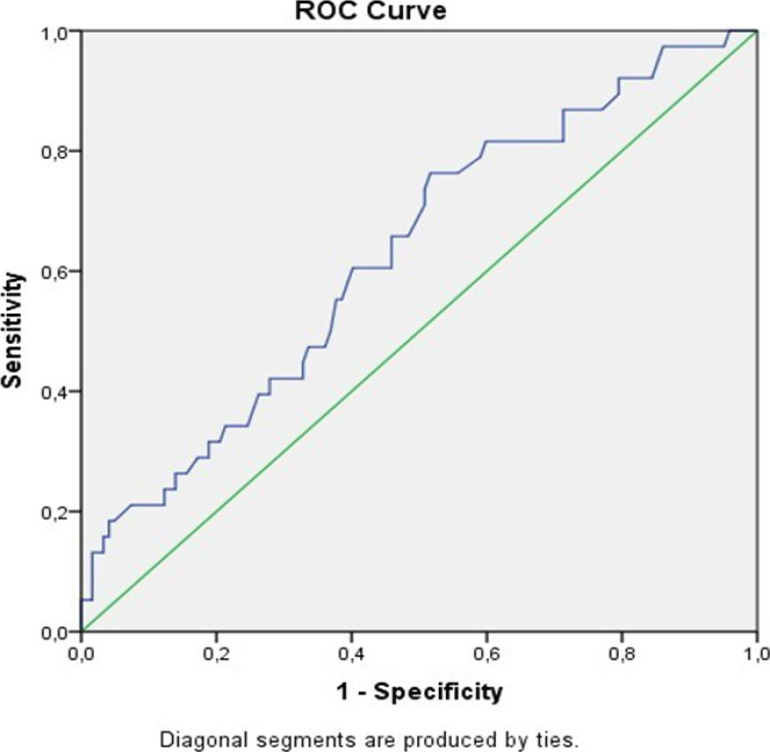
ROC curve evaluating the effectiveness of main pulmonary artery diameter in predicting mortality in COVID-19 patients

**Figure 2:**
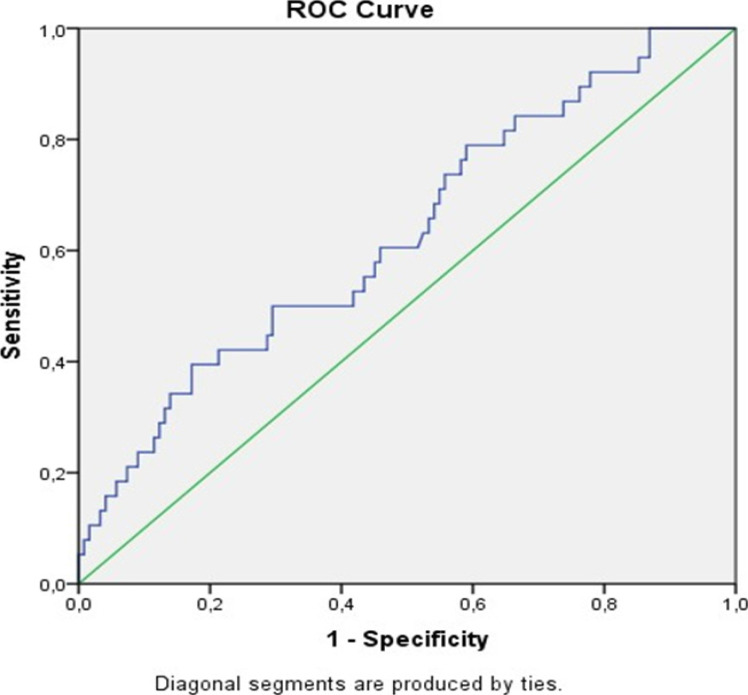
ROC curve evaluating the effectiveness of the main pulmonary artery/ascending aorta diameter ratio in predicting mortality in COVID-19 patients

**Figure 3:**
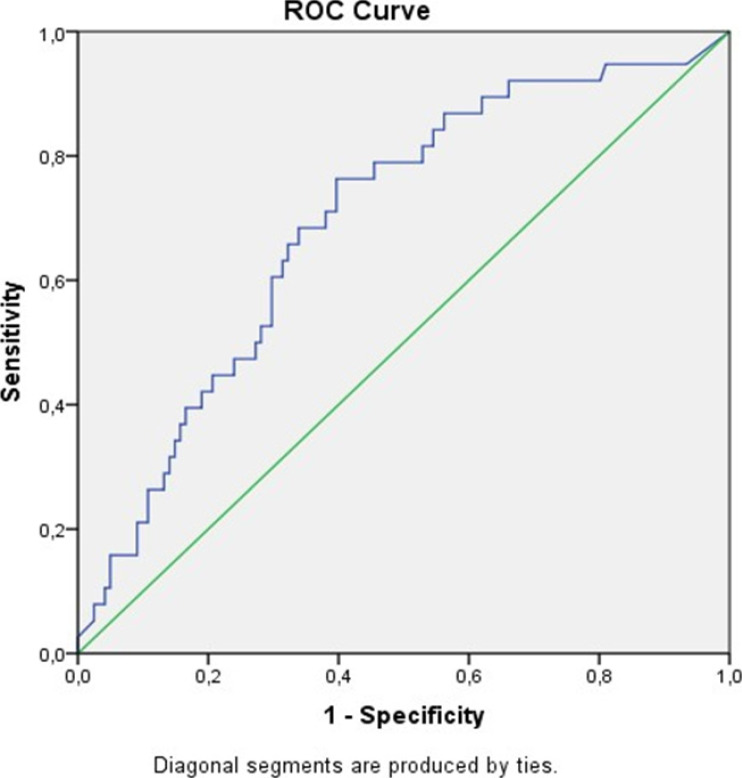
ROC curve evaluating the effectiveness of NT-proBNP level in predicting mortality in COVID-19 patients

ROC curves with 95% confidence interval were produced and cut-off points were determined regarding whether MPA diameter, MPAD/AAD ratio, and NT-proBNP level were predictive factors for mortality in COVID-19 patients. (Figure 1,2,3 and Table 2).

**Table 2:** Best cut-off points for MPA diameter (MPAD), ascending aortic diameter (AAD), and NT-proBNP level that distinguishes the group who died due to COVID-19 from the recovered group with 95% confidence according to the area under the ROC curve

	MPAD (mm)	MPAD/AAD ratio	NT-proBNP
**AUC**	0.624	0.624	0.688
**% 95 CR**	0.515-0.733	0.515-0.733	0.587-0.789
**p value**	0.036	0.036	0.001
**Cut point**	27.5	0.75	742
**Sensitivity**	0.733	0.767	0.733
**Specificity**	0.488	0.421	0.603

**AUC:** area under the curve, **CR:** confidence range, **MPAD:** Main pulmonary artery diameter, **AAD:** Ascending aortic diameter.

According to multivariate logistic regression analysis, advanced age was identified as independent risk factors predicting mortality in COVID-19 patients (Table 3) (respectively, (p=0.005), (p=0.004), (p=0.008), (p=0.002), (p= 0.039))

**Table 3:** Determination of risk factors for mortality in patients with COVID-19 using univariate and multivariate logistic regression analysis

	Univariate			Multivariate	

	**OR (% 95 GA)**	p		**OR (% 95 GA)**	p
			
**Age (years)**	1.046 (1.011-1.081)	**0.009**	**Age (years)**	1.095 (1.027-1.167)	**0.005**
**Hypertension**	3.072 (1.172-8.053)	**0.022**	**Hypertension**	2.036 (0.414-10.008)	0.391
**CAD**	3.29 (1.443-7.554)	**0.005**	**CAD**	7.469 (1.905-29.279)	**0.004**
**Dementia**	9.917 (2.318-42.43)	**0.002**	**Dementia**	13.082 (1.98-86.413)	**0.008**
**Albumin (g/dL)**	0.227 (0.097-0.532)	**0.001**	**Albumin (g/dL)**	0.739 (0.211-2.59)	0.637
**CRP (gr/L)**	1.005 (1-1.01)	**0.045**	**CRP (gr/L)**	1.012 (1.003-1.021)	**0.007**
**D-dimer (mg/L)**	1.189 (1.048-1.35)	**0.007**	**D-dimer (mg/L)**	1.282 (1.092-1.505)	**0.002**
**Procalcitonin (µg/L)**	1.247 (0.881-1.766)	0.213			
**Maximum ferritin level (ug/L)**	1 (1-1.001)	**0.001**	**Maximum ferritin level (ug/L)**	1.001 (1-1.001)	**<0.001**
**Maximum IL-6 level (pg/mL)**	1 (1-1.001)	0.345			
**NT-proBNP > 742 (pg/mL)**	4.24 (1.746-10.293)	**0.001**	**NT-proBNP > 742 (pg/mL)**	3.542 (1.745-9.463)	**0.021**
**Troponin (ng/mL)**	1.001 (0.999-1.002)	0.318			
**MPAD > 27.5 (mm)**	2.492 (1.03-6.031)	**0.043**	**MPAD > 27.5 (mm)**	0.676 (0.166-2.76)	0.586
**MPAD/AAD ratio > 0.75**	2.282 (1.21-5.724)	0.049	**MPAD/AAD > 0.75**	2.692 (1.264-9.312)	**0.036**
**Pleural fluid**	3.669 (1.324-10.167)	**0.012**	**Pleural fluid**	4.758 (1.084-20.892)	**0.039**

**COPD :** Chronic Obstructive Pulmonary Disease, **CAD:** coronary Artery disease, **LDH:** Lactate dehydrogenase, **CRP:** C reactive protein, **IL-6:** Interleukin 6, **NT-proBNP:** N-terminal pro B-type natriuretic peptide **MPAD:** Main pulmonary artery diameter, **AAD:** Ascending aortic diameter, **CT:** Computed Tomography

In the subsequent analysis, statistically significant (r=0.296, p <0.001) positive correlation was found between NT-proBNP level and MPAD. There was no correlation between MPAD and spread, and the number of lung lobes involved (r: -0.683, p=0.425 and r=- r=- 0.062, p=0.436) (Table 4).

**Table 4:** Evaluation of correlation with MPA diameter, NT-proBNP, CT extent, number of involved lobes and hematological parameters in patients with COVID-19

	MPAD		NT-proBNP Level		CT spread finding		Number of lung lobes involved	
	r	p	r	p	r	p	r	p
**D-dimer**	0.231	**0.004**	0.236	**0.004**	-0.014	0.863	<0.001	0.998
**Interleukin 6**	0.144	0.078	0.227	**0.005**	0.017	0.832	-0.01	0.899
**Ferritin**	0.02	0.806	0.084	0.305	0.226	**0.005**	0.225	**0.005**
**CRP**	0.039	0.636	0.104	0.204	0.283	**<0.001**	0.272	**0.001**
Procalcitonin	0.148	0.07	0.395	**<0.001**	0.073	0.372	0.074	0.367
**Sedimentation**	0.044	0.589	0.242	**0.003**	0.19	**0.019**	0.172	**0.034**

## Discussion

With respect to our results, the MPAD/AAD ratio and NT-proBNP levels were determinates as independent risk factors predicting mortality considering the main findings of our study. The MPAD/AAD ratio was detected as an independent risk factor. In univariant analysis, MPA diameter was convenient in predicting mortality, however this estimation was unable to be seen in multivariate analysis. NT-proBNP increases congruous to MPA diameter increases got us take into consideration that these two parameters should be evaluated together pursuant to correlation analysis.

A study by Spagnola et al. revealed that COVID-19 patients showed a higher median PA maximum diameter and median AAD ratio than those measured on previous chest CT scans for any reason except for CAD. In this study, the cut-off value for MPA was found to be 31 mm 31, while Zhu et al. ascertained the cut-off value for PA to be 29 mm 28.

MPAD/AAD > 1, while it was 13.0% in patients with MPAD/AAD.

In another study evaluating the PA diameter, the mortality rate was 22.2% in patients with ≤ 1 32.

In our study, we found the MPA cut-off value to be 27.5 mm and the MPAD/AAD ratio to be 0. 789.The different cut-off values in these studies may be due to race differences or undetected vascular effects.

Similar to the study of Abosamak et al., we did not find a significant relationship between the severity of CT imaging findings of the disease and the diameter of the PA. This may be due to large amounts of microvascular thrombus in the lung invasion, given the high D-dimer level 33.

Damage of the lungs resulting in vascular resistance causes an increase in PA diameter. Again, due to this increased vascular resistance, especially the right ventricle is affected and it causes an increase in NT-proBNP level due to cardiac involvement (34, 35). We also found a significant positive correlation between PA and NT-proBNP in our study.

In conclusion, NT-proBNP indicates hemodynamic stress. It has proven useful for risk stratification in other conditions such as heart failure (HF), pulmonary embolism, and pneumonia. NT-proBNP is frequently found elevated in COVID-19 and has been evaluated as a strong and independent risk factor for mortality. Therefore, its use in patient follow-up may be a guide in predicting the prognosis 22, 36.

For NT-proBNP, <125 pg/ml (<450 pg/ml over 75 years) can be considered normal in cardiac evaluation other than COVID-19 37, 38.

Gao et al. in the study in which they examined the relationship between COVID-19 severity and NT-proBNP levels, the NT-proBNP threshold level determined to predict inhospital mortality was 88.64 pg/mL 39. In our study, an increase in mortality was observed at values above the NT-proBNP threshold value, which was determined as 712 pg/ml. Since this difference may occur due to many reasons, it is clear that more studies are needed on this subject.

In many different studies carried out so far, many parameters such as advanced age, high d-dimer, CRP levels and lymphopenia have been found to be associated with COVID-19 severity 40. Similarly, we found higher levels of CRP, D-dimer, procalcitonin, ferritin, IL-6, NT-proB-NP and troponin and lower albumin levels in patients who deceased.

Laboratory data are more readily available parameters but may not show a specific organ involvement. NT-proBNP is a heart-specific parameter and can guide our treatment. (eg, cardioprotective therapies, need for further cardiac evaluation). Likewise, it is possible to make specific evaluations with CT of thorax. These are the tests that will be needed at any stage of the disease. How much benefit they provide should be updated with the studies done. Advanced age and high d-dimer levels are reported as risk factors for COVID-19. However, it is unknown whether early radiographic change is an indicator for mortality. Advanced age, high LDH level at presentation, and high severity score of CT images within the first week are potential predictors for mortality in adults with COVID-19. These predictors can help clinicians to identify patients with a poor prognosis at an early stage 40, 41.

## Limitations

The lack of pre-COVID-19 PA diameter measurement data of the patients, the lack of follow-up after the disease, the retrospective nature of the study and the relatively small sample size were our important limitations. In addition, the CTs were unenhanced. Arterial diameter measurements in noncontract CT have some difficulties. However, non-contrast CT is preferred in COVID-19 patients for ease of acquisition and to avoid contrast-related complications. Contrast-enhanced CT was not performed as we aimed to evaluate the available data.

As a result, any scientific contribution regarding COVID-19, which has become the most important health problem all over the world, is vital. Since it is an easy measurement and non-invasive procedure, measurement of PA diameter and its evaluation together with NT-proB-NP will provide an additional contribution to the clinician in patient follow-up and predicting the prognosis. We believe that there is a necessity for larger-scale and prospective studies on the subject.

## Conclusions

COVID-19 continues to affect the world with its different clinical presentations and unknowns. As the most important endpoint for the disease is death, it is crucial for the clinician to reveal the factors that predict mortality. Adequate interpretation of easily accessible and non-invasive thoracic CT scan findings in COVID-19 patients will strengthen our hand for the treatment process. It will also benefit from organ-specific blood tests.

In our study, we found MPAD/AAD ratio and NT-proB-NP as independent risk factors in predicting mortality.

## References

[1] Gao Z, Xu Y, Sun C, Wang X, Guo Y, Qiu S (2021). A systematic review of asymptomatic infections with COVID-19. J Microbiol Immunol Infect.

[2] Medicine JHU (2022). Johns Hopkins Coronavirus Resource Center.

[3] Waller JV, Kaur P, Tucker A, Lin KK, Diaz MJ, Henry TS (2020). Diagnostic Tools for Coronavirus Disease (COVID-19): Comparing CT and RT-PCR Viral Nucleic Acid Testing. AJR Am J Roentgenol.

[4] Ye Z, Zhang Y, Wang Y, Huang Z, Song B (2020). Chest CT manifestations of new coronavirus disease 2019 (COVID-19): a pictorial review. Eur Radiol.

[5] Alsharif W, Qurashi A (2021). Effectiveness of COVID-19 diagnosis and management tools: A review. Radiography (Lond).

[6] Malguria N, Yen LH, Lin T, Hussein A, Fishman EK (2021). Role of Chest CT in COVID19. J Clin Imaging Sci.

[7] Miesbach W, Makris M (2020). COVID-19: Coagulopathy, Risk of Thrombosis, and the Rationale for Anticoagulation. Clin Appl Thromb Hemost.

[8] Fox SE, Akmatbekov A, Harbert JL, Li G, Quincy Brown J, Vander Heide RS (2020). Pulmonary and cardiac pathology in African American patients with COVID-19: an autopsy series from New Orleans. Lancet Respir Med.

[9] Yildiz M, Yadigar S, Yildiz B, Aladag NB, Keskin O, Ozer RS (2021). Evaluation of the relationship between COVID-19 pneumonia severity and pulmonary artery diameter measurement. Herz.

[10] Esposito A, Palmisano A, Toselli M, Vignale D, Cereda A, Rancoita PMV (2021). Chest CT-derived pulmonary artery enlargement at the admission predicts overall survival in COVID-19 patients: insight from 1461 consecutive patients in Italy. Eur Radiol.

[11] Truong QA, Massaro JM, Rogers IS, Mahabadi AA, Kriegel MF, Fox CS (2012). Reference values for normal pulmonary artery dimensions by noncontrast cardiac computed tomography: the Framingham Heart Study. Circulation Cardiovascular imaging.

[12] Ieneke JC, Allison's G (2016). Hartmann CMS-P. Pulmonary Circulation and Pulmonary Thromboembolism. Diagnostic Radiology.

[13] Maev IV, Merzlikin LA, Kaziulin AN, Vorob'ev LP (1991). [Doppler echocardiographic assessment of pulmonary blood flow in patients with acute pneumonia]. Ter Arkh.

[14] Light RB (1999). Pulmonary pathophysiology of pneumococcal pneumonia. Semin Respir Infect.

[15] Corrales-Medina VF, Musher DM, Shachkina S, Chirinos JA (2013). Acute pneumonia and the cardiovascular system. Lancet.

[16] Spagnolo P, Cozzi A, Foà RA, Spinazzola A, Monfardini L, Bnà C (2020). CT-derived pulmonary vascular metrics and clinical outcome in COVID-19 patients. Quant Imaging Med Surg.

[17] Hall C (2005). NT-ProBNP: the mechanism behind the marker. J Card Fail.

[18] Yap LB, Mukerjee D, Timms PM, Ashrafian H, Coghlan JG (2004). Natriuretic peptides, respiratory disease, and the right heart. Chest.

[19] Nagaya N, Nishikimi T, Okano Y, Uematsu M, Satoh T, Kyotani S (1998). Plasma brain natriuretic peptide levels increase in proportion to the extent of right ventricular dysfunction in pulmonary hypertension. J Am Coll Cardiol.

[20] Leuchte HH, Holzapfel M, Baumgartner RA, Ding I, Neurohr C, Vogeser M (2004). Clinical significance of brain natriuretic peptide in primary pulmonary hypertension. J Am Coll Cardiol.

[21] Rudiger A, Gasser S, Fischler M, Hornemann T, von Eckardstein A, Maggiorini M (2006). Comparable increase of B-type natriuretic peptide and amino-terminal pro-B-type natriuretic peptide levels in patients with severe sepsis, septic shock, and acute heart failure. Crit Care Med.

[22] Caro-Codón J, Rey JR, Buño A, Iniesta AM, Rosillo SO, Castrejon-Castrejon S (2021). Characterization of NT-proBNP in a large cohort of COVID-19 patients. Eur J Heart Fail.

[23] Jadhav P, Yue B, Guerra MR, Shah N, Haozhe S, Tang H (2021). ELEVATION OF NT-PROBNP LEVEL IS ASSOCIATED WITH HIGHER IN-HOSPITAL MORTALITY FOR COVID-19 IN ETHNIC MINORITY COMMUNITY. Journal of the American College of Cardiology.

[24] Aslam MI, Minhas A, Ghorbani A, Shade JK, Jani V, Hsu S (2021). Natriuretic Peptide Levels and Clinical Outcomes Among Patients Hospitalized with Coronavirus Disease 2019 Infection. Crit Care Explor.

[25] Nair D, Dani S, Masshadi M (2021). Association of N-terminal pro-brain natriuretic peptide with mortality in COVID-19: A systematic review and meta-analysis. Indian heart journal.

[26] Wang L, Chen F, Bai L, Bai L, Huang Z, Peng Y (2021). Association between NT-proBNP Level and the Severity of COVID-19 Pneumonia. Cardiol Res Pract.

[27] Erdoğan M, Öztürk S, Erdöl MA, Kasapkara A, Beşler MS, Kayaaslan B (2021). Prognostic utility of pulmonary artery and ascending aorta diameters derived from computed tomography in COVID-19 patients. Echocardiography.

[28] Zhu QQ, Gong T, Huang GQ, Niu ZF, Yue T, Xu FY (2021). Pulmonary artery trunk enlargement on admission as a predictor of mortality in in-hospital patients with COVID-19. Jpn J Radiol.

[29] Bernheim A, Mei X, Huang M, Yang Y, Fayad ZA, Zhang N (2020). Chest CT Findings in Coronavirus Disease-19 (COVID-19): Relationship to Duration of Infection. Radiology.

[30] Wells JM, Washko GR, Han MK, Abbas N, Nath H, Mamary AJ (2012). Pulmonary arterial enlargement and acute exacerbations of COPD. N Engl J Med.

[31] Spagnolo P, Cozzi A, Foà RA, Spinazzola A, Monfardini L, Bnà C (2020). CT-derived pulmonary vascular metrics and clinical outcome in COVID-19 patients. Quantitative imaging in medicine and surgery.

[32] Eslami V, Abrishami A, Zarei E, Khalili N, Baharvand Z, Sanei-Taheri M (2021). The Association of CT-measured Cardiac Indices with Lung Involvement and Clinical Outcome in Patients with COVID-19. Acad Radiol.

[33] Abosamak MF, Henry BM, Aly MF, Lavie CJ, Sanchis-Gomar F (2021). CT-Determined Maximum Pulmonary Artery to Ascending Aorta Diameter Ratio in Nonsevere COVID-19 Patients. Academic radiology.

[34] Milligan GP, Alam A, Guerrero-Miranda C (2020). Recognizing Right Ventricular Dysfunction in Coronavirus Disease-2019-Related Respiratory Illness. J Card Fail.

[35] Jentzer JC, Mathier MA (2016). Pulmonary Hypertension in the Intensive Care Unit. J Intensive Care Med.

[36] Pranata R, Huang I, Lukito AA, Raharjo SB (2020). Elevated N-terminal pro-brain natriuretic peptide is associated with increased mortality in patients with COVID-19: systematic review and meta-analysis. Postgrad Med J.

[37] Silvers SM, Howell JM, Kosowsky JM, Rokos IC, Jagoda AS (2007). Clinical policy: Critical issues in the evaluation and management of adult patients presenting to the emergency department with acute heart failure syndromes. Ann Emerg Med.

[38] Kistorp C, Raymond I, Pedersen F, Gustafsson F, Faber J, Hildebrandt P (2005). N-terminal pro-brain natriuretic peptide, C-reactive protein, and urinary albumin levels as predictors of mortality and cardiovascular events in older adults. Jama.

[39] Gao L, Jiang D, Wen XS, Cheng XC, Sun M, He B (2020). Prognostic value of NTproBNP in patients with severe COVID-19. Respir Res.

[40] Li K, Chen D, Chen S, Feng Y, Chang C, Wang Z (2020). Predictors of fatality including radiographic findings in adults with COVID-19. Respir Res.

[41] Guzik TJ, Mohiddin SA, Dimarco A, Patel V, Savvatis K, Marelli-Berg FM (2020). COVID-19 and the cardiovascular system: implications for risk assessment, diagnosis, and treatment options. Cardiovasc Res.

